# Effect of Climate on Volatile Metabolism in ‘Red Globe’ Grapes (*Vitis vinifera* L.) during Fruit Development

**DOI:** 10.3390/foods11101435

**Published:** 2022-05-16

**Authors:** Nan Xiang, Hui Xie, Liuwei Qin, Min Wang, Xinbo Guo, Wen Zhang

**Affiliations:** 1 Guangdong Province Key Laboratory for Green Processing of Natural Products and Product Safety, Engineering Research Center of Starch and Vegetable Protein Processing Ministry of Education, School of Food Science and Engineering, South China University of Technology, Guangzhou 510640, China; nanxiang0908@163.com (N.X.); qlw13710614008@163.com (L.Q.); 2 Research Institute of Horticulture, Xinjiang Academy of Agricultural Sciences, Urumqi 830091, China; xhxjnky@163.com (H.X.); wangmin_807032699@163.com (M.W.)

**Keywords:** grape berry, volatile, transcriptome, metabolome, fruit development, climate

## Abstract

With unique flavor and nutritional value, grapes are popular for eating and for the byproducts obtained in their processing. This study cultivated a popular grape variety, ‘Red Globe’, in two regions with different climates to investigate the discrepancies in their volatiles in response to climate. Saccharides, organic acids and transcriptomic and volatile metabolic analyses were studied separately via GC-FID, RNA sequencing and GC-MS/MS methods during the development of grape berries. In total, 83 volatiles were determined in samples, with (E)-2-hexenal the most abundant. Fatty acid derivatives and terpenoids in grapes showed discrepancies in different climates, and some of them were correlated to specific transcription factors. *VvWRKY22* was influenced by climate conditions and was relative to saccharide accumulation. MYB-related transcription factors (TFs) were highly correlated with volatiles that accumulated during fruit ripening, especially decanal. Terpenoids showed correlations with a gene module that contained *ERFs* and *HSFs*. The findings support the hypothesis that fruit maturity and volatile formations vary in grape berries under different climates. Moreover, specific TFs could participate in volatile accumulations. The given results not only serve to enrich theoretical knowledge on the regulatory mechanism of volatiles in grapes, but also provide guidance for enhancing grape flavor and aroma by modulating cultivational conditions.

## 1. Introduction

As commercial fruits with popular flavor, grapes (*Vitis vinifera* L.), are widely planted in arid and semi-arid areas [1]. They possess high nutritional value owing to the rich contents of carbohydrates, organic acids, and minerals [2]. Apart from their everyday use as a fresh fruit for eating, grapes can be processed into a number of other products, such as raisins, wine and juice, in which flavor is one of the most important commercial indexes. Therefore, analyzing grape volatiles, which are the predominant contributors to the fruity notes and flavors of grape products [3], is necessary.

Grape volatiles, derived from several pathways and belonging to different chemical groups, are multifarious and vary from species to species [4]. Specifically, the varietal volatile profiles of grapes are constituted by monoterpenoids, sesquiterpenoids and C_13_ norisoprenoids [5]. The bound aroma fraction in most grape varieties does not directly provide the flavor. On the contrary, crushing or processing on grapes transforms their volatiles into odor-active forms [6], including C_6_ compounds, which can provide wine with a strong herbaceous flavor [7], terpenes, C_13_ norisoprenoids, benzenoid compounds, and aliphatic alcohols in the skin of grapes [3]. Moreover, aroma molecules could be formed by salivary or bacterial enzymes from two odorless precursors, glutathionyl and cysteinyl [8]. Consequently, the flavor of grape products is affected by free aroma fraction embedded in fresh grapes combined with aroma production and transformation during processing and consumption.

Growing in China, grapes in different regions enjoy various cultivational environments. Some volatiles are reported as plant secondary metabolites and their syntheses are easily affected by climate conditions [9]. As investigated, low water treatment in ‘Cabernet Sauvignon’ grapes produced desirable aromas that differed from the unfavorable herbaceous-related aromas induced by irrigation treatment [10]. Comparing the volatile profiles of grapes in different environmental conditions, terpenoids were higher in samples that grew at high altitude and room temperature (25 °C) [5]. Warm conditions could preserve more monoterpenes than cool conditions in different grape varieties from several countries [11]. In regions with higher temperature and humidity, norisoprenoids were more abundant in grapes [11]. Furthermore, (E)-β-damascenone, β-ionone, hexanal, 2-hexanol, (Z)-3-hexenol and (E)-2-hexenol were highly correlated with various environmental parameters [11].

Moreover, volatiles are regarded as stress-responsive compounds in plants. For example, both long-term heat and heat-shock stresses were shown to enhance the elicitation of lipoxygenase and glucosinolate volatiles in *Brassica nigra* [12]. A group of specific volatiles, including mono- and sesquiterpenes, were found to be stress-responsive and played important roles in plant stress tolerance [13]. Recently, several studies indicated that the stress-responsive roles of volatiles were modulated by groups of TFs, which expanded our knowledge on the modulative mechanism of volatiles under stress. Previously, transcription repressor TPLs were proved to mediate volatile organic compounds in tobacco [14]. In tea, low temperature combined with wounding stress stimulated CsMYC2, which was involved in jasmonic-acid signaling, thus facilitating the biosynthesis of indole [15].

As noted, volatile profiles in grapes are dependent on growing conditions and are stress-responsive. However, how grape volatiles are regulated by surrounding conditions has been less comprehensively studied. Therefore, we selected grapes grown in two regions with various climate conditions in China to investigate the diverse composition of their volatiles. Furthermore, the present study combined transcriptomic analyses to identify key genes as well as TFs, aiming to understand the inner regulative mechanism of volatiles, and hence could improve grape plantations as well as byproduct quality.

## 2. Materials and Methods

### 2.1. Sample Collection and Growth Environment

The ‘Red Globe’ (*Vitis vinifera* L.) grapes selected for investigation were separately cultivated at the experiment bases of Xinjiang Institute of Agricultural Sciences in Turpan and Urumqi, China. The cultivated environment was reported in a recent study [2]. In brief, during fruit development in Turpan, the average temperature was about 40 °C, average humidity was about 40% and light intensity was around 1500–1800 μmol·m^−2^·s^−1^. By contrast, growing conditions in Urumqi included an average temperature of about 30 °C, average humidity of about 50% and light intensity of around 900–1200 μmol·m^−2^·s^−1^. In contrast to the temperate continental climate of Urumqi, Turpan possesses an arid, warm continental climate owing to its basin topography with longer sun exposure time. Fifteen grapevines with the same ages and growth trends that had been cultivated at the experimental bases of each district were selected for experimental use. At each stage, a bunch of grapes with similar growth trends from each grapevine was selected for sample collection. Five to ten well-developed berries without worms were collected from each of the bunches. Collected berries were divided into three parts for three replicates. Then the berries were immediately frozen by liquid nitrogen and stored at −80 °C until use. Berries collected at 45 days after flowering were labelled in accordance with their collection time as Stage 1 (S1). Then, berries were collected at intervals of fourteen days and labelled as Stages 2 to 5 (S2 to S5). External profiles of the selected grape berries are shown in Figure S1. Samples grown in Turpan are labelled as TS, and samples growing in Urumqi are labelled as US.

### 2.2. Determinations of Saccharides and Organic Acids

The saccharide and organic acid contents were analyzed by GC-FID and quantified with an internal standard method [16]. Samples weighing 5 g were extracted by 40 mL distilled water. After centrifugation, supernatant was collected and filtered by 0.45 μm filter. D-xylose and succinic acid were separately used as internal standards for the qualifications of saccharides and organic acids, which were mixed with extracts and dried by nitrogen and phosphorus pentoxide. Hexamethyldisilazane/trimethylchlorosilane/pyridine (2:1:10, V/V/V) was then added for dissolution and detection. Agilent 6890 N network GC system with DB-1 chromatographic column and Agilent 7683 autosampler were used for determination. Values were presented as mean ± SD mg·g^−1^ fresh weight (FW) (*n* = 3).

### 2.3. Volatile Analysis

The determination was processed in a gas chromatography–triple quadrupole mass spectrometer (GC-MS/MS) as previously reported [17]. Grape berries were immediately frozen by liquid nitrogen and powdered by grinding instrument. Subsequently, 2 g of this powder was weighed into a headspace bottle for detection. Volatiles were processed according to the previously reported program and extracted by a 15 μm PDMS/DVB extraction fiber (ANPEL, Shanghai, China). After being separated by GC system, compounds underwent splitless injection for the analysis. A mass spectrogram of each compound was compared to the NIST 2020 library (Aglient Technologies G1033A, Santa Clara, CA, USA) for qualification. L-2-octanol was purchased from Shanghai Yuanye Bio-Technology (Shanghai, China) and was added into the headspace bottle as an internal standard for semi-quantification. Results were exhibited as ng·kg^−1^ FW (*n* = 3).

### 2.4. RNA Sequencing

Grape berries were quickly frozen by liquid nitrogen and stored at −80 °C. Samples were sent to Beijing Genomics Institution (BGI, Beijing, China) for RNA extraction, construction of the cDNA corpus and RNA sequencing. In brief, the total RNA was enriched by the mRNA enrichment method, as previously reported [17]. The connected products were amplificated and cyclized to construct the cDNA corpus for sequencing.

### 2.5. The Validation of TFs

The validation of TFs was conducted by the real time quantitative PCR (RT-qPCR) method. Summarily, the extracted RNA from the grapes was reversed to cDNA by FastKing gDNA Dispelling RT SuperMix Kit (Takara Biotechnology, Dalian, China) and RT-qPCR was completed with a SuperReal PreMix Plus Kit (Tiangen, Beijing, China) in a CFX96 Real-Time PCR System (Bio-Rad Laboratories, Inc., Hercules, CA, USA). *VvUBI* was used as a reference gene. Primers used in the present study were listed in Table S1. Relative expressions of genes were calculated with the 2^−ΔΔCt^ method according to Ct value, as previously used [17]. Results were expressed as mean ± SE in triple.

### 2.6. Weighted Gene Co-Expression Network Analysis (WGCNA)

WGCNA was operated according to the previously published method [18]. The different expression genes (DEGs) filtered between US and TS at each stage were analyzed. The screened FPKM was set as 0.1. The similarity threshold value of module fusion was set as 0.5. The minimum gene number in a module was 30 in order to maintain a high reliability of results.

### 2.7. Statistical Analysis

Heatmap cluster, time cluster pattern, principal component analyses and fold change analyses were performed by MetaboAnalyst 5.0. Figures were depicted in Origin 2018 and an online website: http://www.bioinformatics.com.cn (accessed on 16 August 2021). with modification. The Tukey method was conducted for analyzing significant differences (*p* < 0.05) and the Pearson correlation method was performed for correlation analysis on IBM SPSS Statistics 25 (IBM Corp., Armonk, NY, USA). Samples were collected with three parallels, and the results of the gene validations were expressed as mean ± SE, while others were exhibited as mean ± SD.

## 3. Results

### 3.1. Saccharides and Organic Acids

Two monosaccharides and three organic acids, including fructose, glucose, malic acid, tartaric acid and citric acid were identified and quantified by GC-FID, as presented in Table 1. With the elongation of ripening time, saccharides were accumulated, and correspondingly, acids were reduced in both TS and US. Among them, citric acid was only detected in S1 and was lost in the following growing period. Interestingly, saccharides dramatically increased and organic acids sharply declined in US from S1 to S2. However, there were comparatively mild trends in the accumulation of saccharides and the reduction of organic acids in TS.

### 3.2. Volatile Profiles

In total, 83 kinds of volatiles were determined by GC-MS/MS in grapes, including phenylpropanoid/benzenoid derivatives; terpenoids; fatty acid derivatives, such as alcohols, aldehydes and esters; and so on. Table S2 numbers these compounds and lists their retention time and structures, while contents can be seen in Table S3. The 83 volatiles are clustered and shown in Figure S2. As depicted, TS samples from S3 to S5 were clustered to US samples at S4 and S5, whereas the remainder were clustered. Examining the outcome in Table S3, (E)-2-hexenal was the most abundant composition in the samples, followed by 2,5-dimethylbenzaldehyde and hexanal. Undecane and indole were separately detected in US at S2 and in TS at S4, whereas α-terpineol, phytol and junenol existed only in US at S1. Furthermore, 3-methylpentane, β-myrcene, dihydrocarveol, styrene, (E,Z)-2,6-nonadienal and azulene could only be detected in TS, while naphthalene and coumaran were unique to US. The majority of those differential volatiles were fatty acid derivatives and terpenoids.

**Figure 1:**
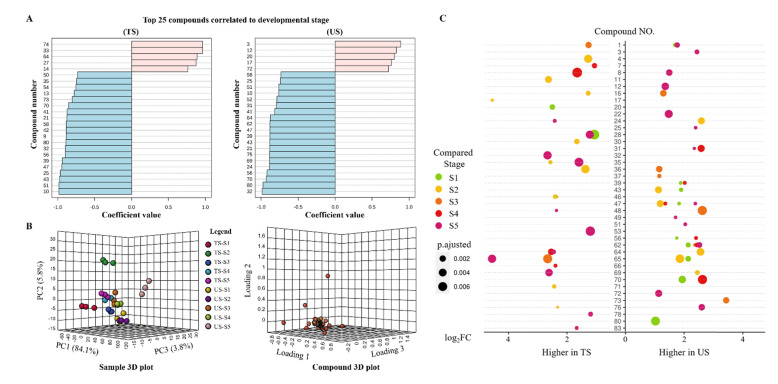
Volatile profiles of grape berries. Compounds are labeled with numbers consistent with Tables S2 and S3. (**A**): The top 25 compounds correlated to developmental stages. (**B**): 3D plot of principal component analysis; (**C**): Significantly varied compounds between US and TS at each stage (FC > 2, *p* < 0.01). TS: Turpan sample; US: Urumqi sample.

The time-accumulating patterns of volatiles from S1 to S5 in both TS and US grapes were determined and the top 25 positively and negatively correlated compounds are listed in Figure 1A. A minority of accumulated volatiles in TS and US were classified into fatty acid and phenylpropanoid/benzenoid derivatives. In TS, (E,Z)-2,6-nonadienal was absent in primary stages but gradually increased in the following periods. 4,6-Dimethyldodecane, 1-hexanol and 2,4-di-tert-butylphenol increased almost three-fold, six-fold and two-fold, respectively, from S1 to S5 in US. However, the majority of compounds were gradually reduced during fruit ripening. The same decline patterns were exhibited by 13 volatiles in both US and TS; 7 of these were terpenoids, and (E)-2-hexenal was included. Notably, during fruit ripening, 3-hydroxy-4-methoxybenzonitrile was constantly accumulated in TS but gradually reduced in US. The varying levels of volatiles growing in different regions might contribute to the variations in grape flavors.

Principal component analysis (PCA) was also conducted. As exhibited in Figure 1B, PC1, PC2 and PC3 separately explained the variations of 84.1%, 5.8% and 3.8%, respectively, totaling 93.7%. (E)-2-hexenal ranked first in PC1 as absolute coefficient value 0.982. 1-Hexanol held the highest coefficient value 0.865 in PC2, while the coefficient value of 2,5-dimethylbenzaldehyde was 0.621 in PC3. Besides, except for TS-S1, TS-S2 and US-S5, other samples were localized in plots within close range of each other. Together with the cluster results in Figure S2, the mixture of TS and US in the volatile profiles is probably due to their similar maturity.

Figure 1C depicts the significant differences in compound contents between US and TS at each stage (fold change (FC) > 2, *p* < 0.01). Notably, 1-hexanol was around 23-fold higher in TS than in US at S2. At all stages, the contents of decanal and 2,6,10,15-tetramethylheptadecane in TS were generally higher than in US. US had the higher nonadecane content at early stages, while TS possessed more nonadecane at later stages. Apart from the above components, low contents of terpenoids such as α-cadinene, δ-amorphene, α-muurolene, α-calacorene and linalool were detected in TS.

### 3.3. Transcriptomic and TF Analyses

In order to understand the effects of climate on grape volatiles at transcriptional levels, the RNA sequencing method was conducted on grape samples. After raw quality filtering, the results comprised about 185.73 Gb of clean sequence data with 40 to 45 million clean reads of each sample. Altogether, 77.33% to 91.23% reads were successfully mapped on *Vitis vinifera* reference genome (GCF_000003745.3_12X) via HISAT. Basic information of RNA sequencing was listed in Table S4.

**Figure 2:**
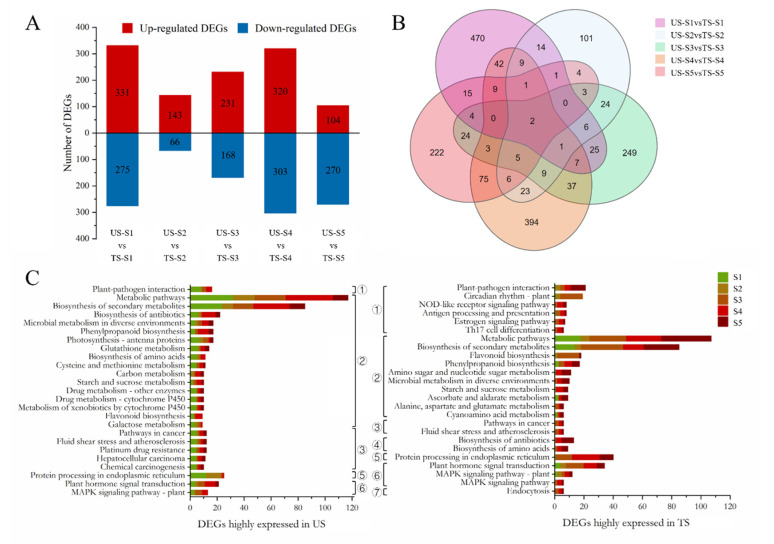
Transcriptomic profile of grape berries. (**A**): Comparison of the significantly up- and downregulated DEG numbers between TS and US at each stage (FC > 2, *p* < 0.01). (**B**): Venn diagrams of DEGs between TS and US at each stage. (**C**): KEGG classification of DEGs between TS and US at each stage. All the pathways were classified into seven categories, including (1): Organismal Systems; (2): Metabolism; (3): Human Diseases; (4): Global and Overview Maps; (5): Genetic Information Processing; (6): Environmental Information Processing; (7): Cellular Processes. TS: Turpan sample; US: Urumqi sample.

The analyses of DEGs were completed in TS and US at each stage. Results are shown in Figure 2A (FC > 2, *p* < 0.01) and are listed in Table S5. The most abundant DEGs were found at S4, whereas the fewest DEGs were witnessed at S2. The Venn graph depicts DEGs at all five stages (Figure 2B). Chaperone protein DnaJ (GenBank ID: 100261077) and desiccation-related protein PCC13-62 (GenBank ID: 100252547) were diversely expressed in TS and US at all stages. RT-qPCR verification were included to ascertain their expressional profiles, and results are listed in Table S1. As shown, the expressional value of *DnaJ* in TS was higher than that in US at S4, but it was upregulated in US at other stages as compared to TS. Validated by RT-qPCR, the expressions of *PCC13-62* in US were higher than in TS from S2 to S4.

Additionally, DEGs were enriched by the KEGG pathway and divided into seven primary categories, in which the top 25 pathways are depicted in Figure 2C. A total of 906 DEGs were highly expressed in US, while 846 DEGs were upregulated in TS (FC > 2, *p* < 0.01). The two groups of DEGs were annotated into 192 and 206 pathways, respectively (Table S6). As classified, the majority were involved in metabolic pathways and biosynthesis of secondary metabolite categories. In contrast to those in US, the DEGs highly expressed in TS were also related to circadian rhythm and endocytosis. Conversely, DEGs that were upregulated in US were partly enriched into cytochrome P450.

### 3.4. Verification of TFs

**Figure 3:**
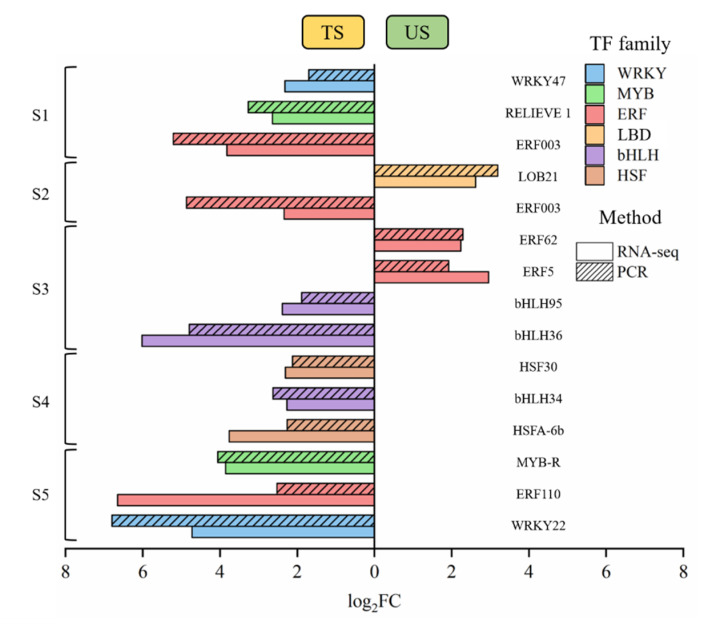
The validation of TFs at specific stages. Genes that expressed more highly in one of the samples (TS or US) are depicted on that side.

KEGG enrichment of TFs was carried out in DEGs at each stage, and then 14 TFs that had significantly differently expressed in TS and US at each stage were selected and verified by RT-qPCR. The comparisons of the RNA sequencing and RT-qPCR results are reported in Figure 3, and detailed expressional profiles are listed in Table S1. In addition, the Pearson correlation was carried out on TFs and volatiles (Table S7). Results showed that at S2, the expressional value of *LOB21* in US was higher than that in TS. Among the five ripening stages, *LOB21* was generally highly expressed in US. The expressional values of both *ERF62* and *ERF5*, from the ERF family, were higher in US than in TS at S3. Additionally, the results of RNA sequencing and RT-qPCR found upregulations of *VvWRKY22*, *HSF30* and *HSFA* in TS at later stages. Furthermore, three TFs from bHLH family showed their high expressional values in TS at S3 or S4.

## 4. Discussion

### 4.1. Saccharides and Organic Acids Indicated Grape Maturity

Glucose and fructose are the major saccharides, while malic acid and tartaric acid account for most of organic acids in grapes [19]. The saccharide and organic acid compositions of ‘Red Globe’ grapes in our study were similar to most varieties of grapes. Previously, researchers found that saccharide content was lower in immature berries and increased during grape ripening, a process accompanied by a decrease in organic acid content [20]. Thus, the discrepancies found between TS and US indicate the influence of climate on grape maturity. Zha et al. [21] inferred that photosynthetic efficiency was directly proportional to light intensity, and could be enhanced by a certain range of daytime temperature. Therefore, grape berries grown in Turpan with high temperature and strong light intensity tend to grow faster than those in Urumqi.

### 4.2. Climate and Fruit Growth Resulted in the Discrepancies in Volatiles in Grapes

Grape volatiles have been widely studied and classified into four groups according to metabolic pathways, including the derivatives of amino acids, phenylpropanoids/benzenoids and fatty acids, as well as terpenoids [22]. As recorded, terpenoids are the most important volatiles for grape flavor, and the monoterpene subtypes are the most abundant volatiles in grape berries [22]. Similarly to the former study [22], fruit ripening changed the terpenoid composition in ‘Red Globe’ grapes in our study. However, although (E)-2-hexenal has previously been detected as a principal component in grapes, it accumulated during fruit ripening [23], in contrast to the decreasing patterns of (E)-2-hexenal in TS and US in our result. The diverse patterns might be influenced by the growth environments, as the previous studied grapes were cultivated in Northern China, where they enjoyed the temperate continental monsoon climate. Incidentally, as it emits a green leafy flavor [23], the decline in (E)-2-hexenal during ripening in the present study might improve the edible flavor of ‘Red Globe’ grapes. Apart from the consequences of ripening, volatiles also changed according to growing conditions. A previous experiment conducted by bagging grapes with different cover colors during ripening periods, indicated the influence of light conditions on decanal [24]. Thus, the higher decanal content in TS than in US indicated its light-enhanced characteristic in the present work. Similarly, terpenoids in tea may be subject to a hypothetical regulatory mechanism, as mature miRNAs maintained by light regulators may negatively regulate terpenoid biosynthesis [25]. Hence, in our results, the consistently lower contents of terpenoids in TS, such as α-cadinene, δ-amorphene, α-muurolene and α-calacorene, might indicate the effects of negative regulation by light intensity.

### 4.3. Principal Genes and TFs

The chaperone protein DnaJ (HSP40) family was shown to have a unique function under heat stress in *Arabidopsis* [26]. *PCC13-62*, as a gene that encodes desiccation-related proteins (DRPs), was less highly expressed in desiccation-sensitive species when compared with desiccation-tolerant species [27]. Therefore, in our study, the different expressional patterns of the two thermal and moisture-related genes in grapes that were cultivated with different average temperatures and humidity levels could enhance existing knowledge on their functions in plant physiological processes.

As mentioned with regard to TFs, a novel essay demonstrated that VvWRKY22 could regulate saccharide accumulation in grapes by interacting with VvSnRK1.1 or VvSnRK1.2 proteins [28]. Moreover, it was reported that saccharides in grapes were influenced by the surrounding conditions during fruit development [29]. As detected, the elongation of actual sunshine duration enhanced the accumulation of saccharides [29]. In our results, the saccharide contents in TS were higher than in US (Table 1); correspondingly, *VvWRKY22* was highly expressed in TS at later stages. Therefore, we inferred that the high expressional pattern of *VvWRKY22* in TS was the outcome of high light intensity and long sunshine duration, and consequently induced the accumulation of saccharides.

Evidently, bHLH34 may bind to the promoter region of *AtPGR* and act as a positive regulator of glucose [30]. Additionally, the upregulation of *SlbHLH95* in tomato fruit ripening resulted in the accumulation of glutathione, soluble saccharides and starch [31]. These results, together with the finding that a higher accumulation of saccharides occurred in grapes from a region with longer sunlit time [29], indicate that bHLHs in grapes probably play a contributary role in saccharide accumulation. Hence, the long sunlit time in Turpan contributed to the high expressional value of bHLHs in TS grapes and modulated saccharide accumulation. In addition, *bHLH95* exhibited a negative correlation (r = −0.975) with (E)-2-hexenal (Table S7), which indicated a possible relationship between the syntheses of (E)-2-hexenal and saccharides.

In plants, *MYB* genes are responders under abiotic stresses, such as drought and cold, and they also participate in light-sensing [32]. Azuma et al. detected that the expressions of three *MYB-related* genes differed dramatically among various light and temperature applications during grape development [33]. Interestingly, the correlation results (Table S7) showed a high correlation value (r = 0.974) between *REVEILLE 1* and decanal in TS. Moreover, *MYB-R* possessed high correlation values with the volatiles that accumulated during fruit ripening in TS (Table S7). Thus, the expressions of *MYB-related* genes in our study might have been enhanced by the combination of stresses from high temperature, high light intensity and low humidity in Turpan region, thus modulating the biosynthesis of volatiles. In general, aside from the previous findings on the regulatory roles of *MYB-related* genes on anthocyanin and flavonoid biosynthesis in grapes [33], we investigated the potential regulatory roles of *MYB-related* genes on grape volatile syntheses.

As reported, heat or drought stress could increase the expressional levels of grape *ERF* genes [34]. Thus, in our study, the high expressions of *ERF003* and *ERF110* might be induced by high temperature and low humidity. Furthermore, HSF30 in grapes might be a key regulator for heat stress and heat recovery [35], hence the two *HSFs* selected in our study were probably stressed by high temperature and consequently upregulated. Overall, these results could enrich the regulatory function of TFs on volatile metabolism in grapes.

### 4.4. WGCNA Analyses on Volatiles and Gene Modules

**Figure 4:**
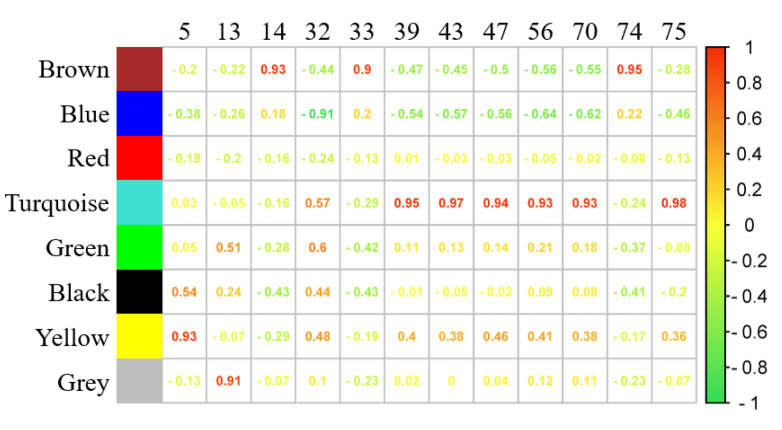
The high correlation among gene modules and several volatiles from WGCNA results. Colors stand for different gene modules. Compounds are labeled by numbers consistent with Tables S2 and S3.

The present work performed WGCNA on the filtered DEGs and detected volatiles, in order to seek relationships between volatile production and gene expression. From a total of 1785 DEGs, 1299 DEGs screened with average FPKM 0.1 are clustered into eight modules (Figure S3 and Table S8). The correlation results are shown in Figure S4 and the prominent correlations were depicted in Figure 4.

In Figure 4, undecane is similarly varied with the yellow module. Genes in this module were enriched into several KEGG pathways, including protein processing and glycosphingolipid biosynthesis (Figure S5A). Styrene, correlated with the grey module (0.91), was degraded from ethylbenzene via the catalyzation of naphthalene 1,2-dioxygenase. Since this step has not been identified in grapes, the relative genes in the grey module might enrich the knowledge of the styrene-generated pathway in grapes. Genes in the brown module were expressed similarly to 2-octanone and (E,Z)-2,6-nonadienal. As depicted in Figure S5B, phytohormones might be involved in their production. KEGG classification exhibited an enrichment in hormone signal transduction, which raises the possibility of these components as external chemical regulators.

Notably, the turquoise module, correlated with several sesquiterpenoids, including α-cadinene, α-muurolene and α-calacorene, with correlated values ranging from 0.93 to 0.98, comprised the genes that enrich photosynthesis (Figure S5C), hence providing evidence for the light regulation of sesquiterpenoids. Notably, those sesquiterpenoids were consistently more abundant in US than in TS. Previously, a study concerning light intensity with regard to *Salvia dolomitica* showed a decline in δ-cadinene in samples exposed to high light intensity [36]; thus, it can be inferred that sesquiterpenoid accumulation in grape berries might be influenced by light intensity. On the other hand, transcription factors *ERF110*, *ERF5*, *HSF30* and *HSFA* were included in the turquoise module. Therefore, the above TFs are assumed to respond toward light conditions and might regulate terpenoid accumulation in grape berries.

## 5. Conclusions

To conclude, in this study, metabonomic and transcriptomic analyses were conducted on grape berries growing in two different climates. Results showed changes in saccharides, organic acids and volatiles during berry ripening, and indicated the discrepancies in the contents of these compounds resulting from distinct climates. The high saccharide and low organic acid contents in TS indicated its greater maturity compared to US. (E)-2-Hexenal was the principal component in the ‘Red Globe’ grape variety, and declined during fruit ripening. We inferred that the high temperature and long sunlight duration in Turpan enhanced the expression of *VvWRKY22*, thus resulting in the abundant saccharides. During the TS fruit ripening, *MYB-related* TFs were highly correlated to decanal and other volatiles that accumulated during fruit ripening. Terpenoid levels in grape berries were probably influenced by light and temperature by *ERF* and *HSF* modulation. The obtained results may enhance understanding of regulatory mechanisms of volatiles in grape berries.

## Figures and Tables

**Table 1:** The saccharide and organic acid contents (mg·g^−1^ FW) in grape berries during the five developmental stages in Urumqi (US) and Turpan (TS).

Site	Period	Fructose	Glucose	Malic Acid	Tartaric Acid	Citric Acid
US	S1	2.52 ± 0.27 d*	12.88 ± 3.75 c	21.58 ± 1.53 a	18.28 ± 1.68 a	1.41 ± 0.21 a
	S2	27.68 ± 1.86 c	37.05 ± 2.87 b	4.51 ± 0.51 c	10.49 ± 0.75 bc	ND
	S3	53.19 ± 3.64 a	59.36 ± 5.85 a	2.52 ± 0.22 de	6.53 ± 0.30 e	ND
	S4	56.41 ± 6.28 a	62.36 ± 6.90 a	1.65 ± 0.22 e	6.26 ± 0.32 e	ND
	S5	53.60 ± 2.62 a	58.49 ± 4.74 a	2.06 ± 0.31 e	6.11 ± 0.14 e	ND
TS	S1	27.94 ± 1.23 c	36.86 ± 0.35 b	6.22 ± 0.49 b	11.48 ± 1.47 b	0.51 ± 0.07 b
	S2	39.66 ± 3.43 b	50.49 ± 4.46 ab	3.70 ± 0.33 cd	8.95 ± 0.35 cd	ND
	S3	49.45 ± 3.47 ab	51.49 ± 7.80 ab	2.44 ± 0.44 de	6.59 ± 0.52 e	ND
	S4	53.03 ± 2.98 a	61.93 ± 5.33 a	1.91 ± 0.15 e	6.69 ± 0.39 de	ND
	S5	59.44 ± 7.28 a	65.06 ± 6.53 a	2.03 ± 0.09 e	6.34 ± 0.59 e	ND

* Different letters stand for significantly differences in each column (*p* < 0.05). FW: fresh weight; S: stage; ND: none detected.

## Data Availability

Not applicable.

## Supplementary Materials

The following supporting information can be downloaded at: https://www.mdpi.com/article/10.3390/foods11101435/s1, Figure S1: External profiles of selected grape berries. DAF: days after flowering. Figure S2: Heatmap and clusters of 83 volatiles in grape berries. Samples are labeled as ‘TS-S1′ to ‘TS-S5′ and ‘US-S1′ to ‘US-S5′, standing for grapes grown in Turpan or Urumqi (TS or US) and development stages (S1 to 5). Figure S3: Cluster dendrogram. Figure S4: The correlation value among gene modules and volatiles. Volatiles were labeled by numbers consistent with Tables S2 and S3. Figure S5: KEGG enrichment of three gene modules. Table S1: Sequences and relative expression of validated genes (*n* = 3). Table S2: Detected volatiles and their structures. Table S3: Information of detected volatiles (ng·kg^−1^). Table S4: Alignments of RNA-sequencing reads. Table S5: Differentially expressed genes. Table S6: KEGG classification of DEGs. Table S7: The correlation values among transcription factors and volatiles.; Table S8: Corresponding modules of analyzed genes.
