# Supplementary material for: Effect of Climate on Volatile Metabolism in ‘Red Globe’ Grapes (Vitis vinifera L.) during Fruit Development
Source: Foods. 2022 May 16;11(10):1435. doi: 10.3390/foods11101435 (PMC9140514; doi:10.3390/foods11101435)
Supplement: Supplementary file 1 [file foods-11-01435-s001.zip › Figures S1-S5.pdf]

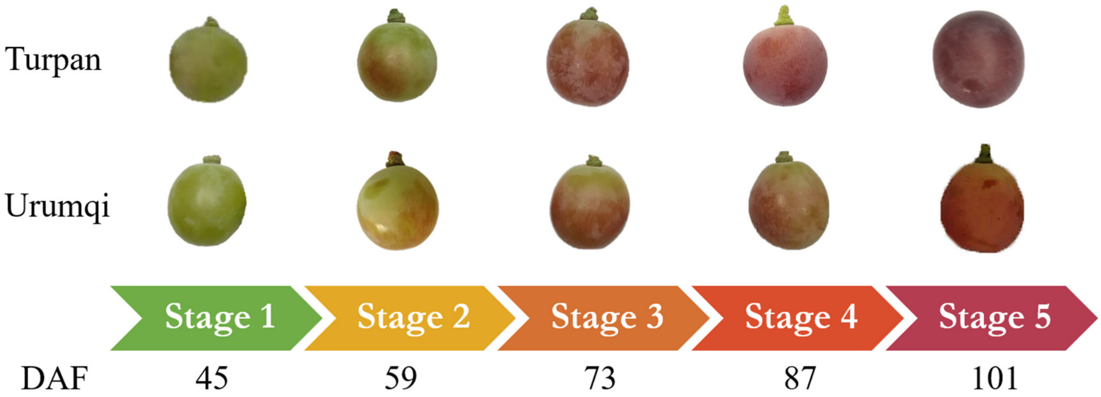

Figure S1. External profiles of selected grape berries. DAF: days after flowering.

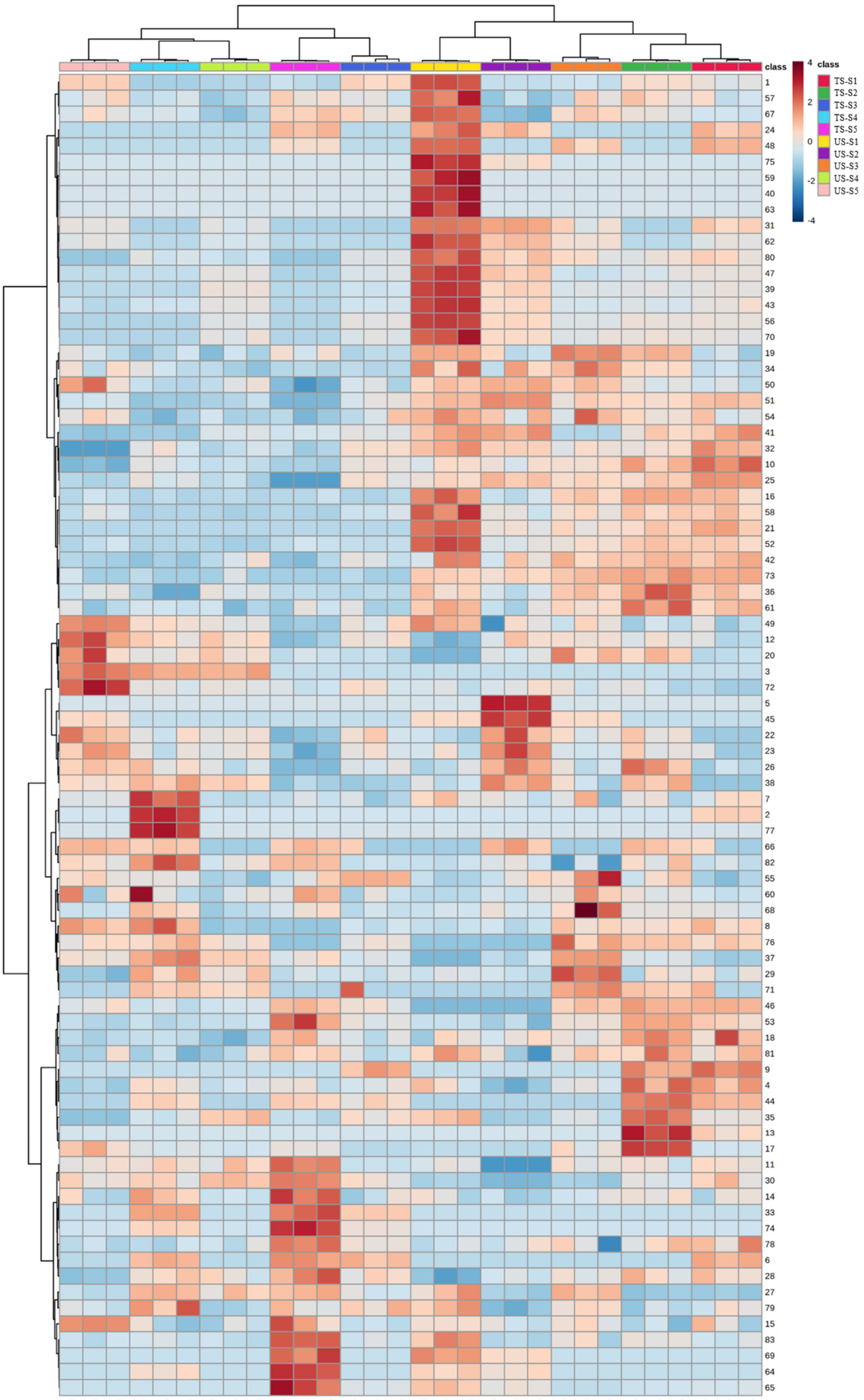

Figure S2. Heatmap and clusters of 83 volatiles in grape berries. Samples are labeled as 'TS-S1' to 'TS-S5' and 'US-S1' to 'US-S5', standing for grapes grown in Turpan or Urumqi (TS or US) and development stages (S1 to 5).

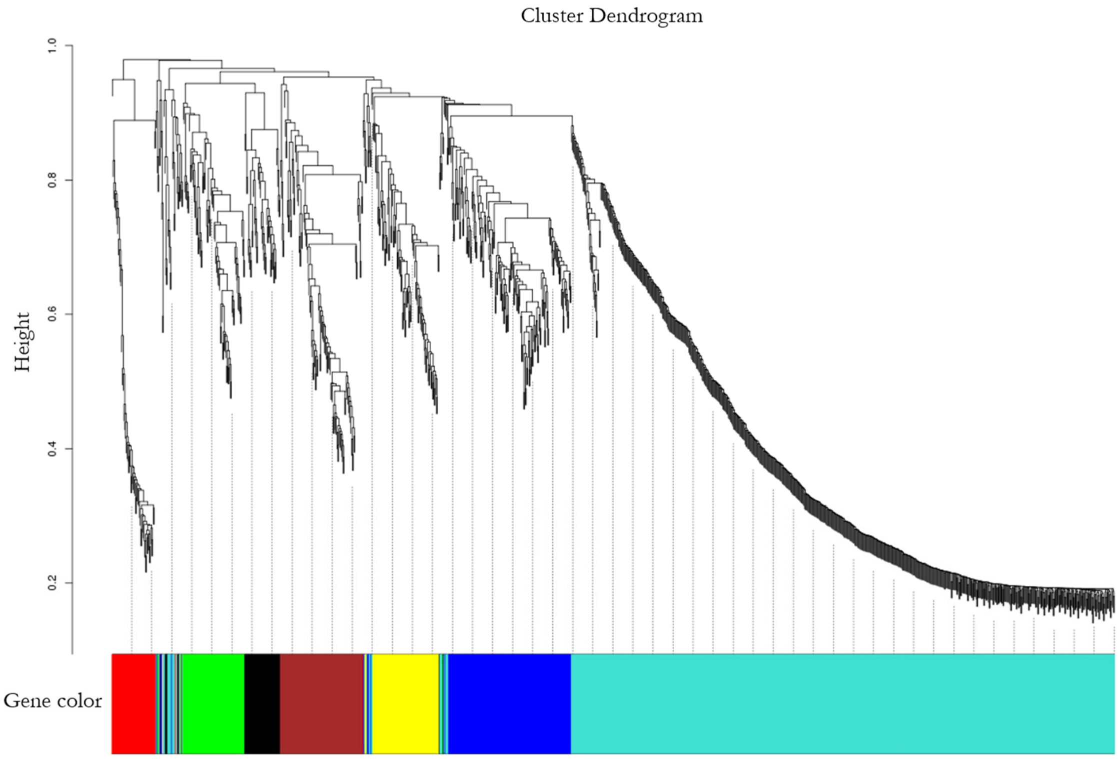

Figure S3. Cluster dendrogram.

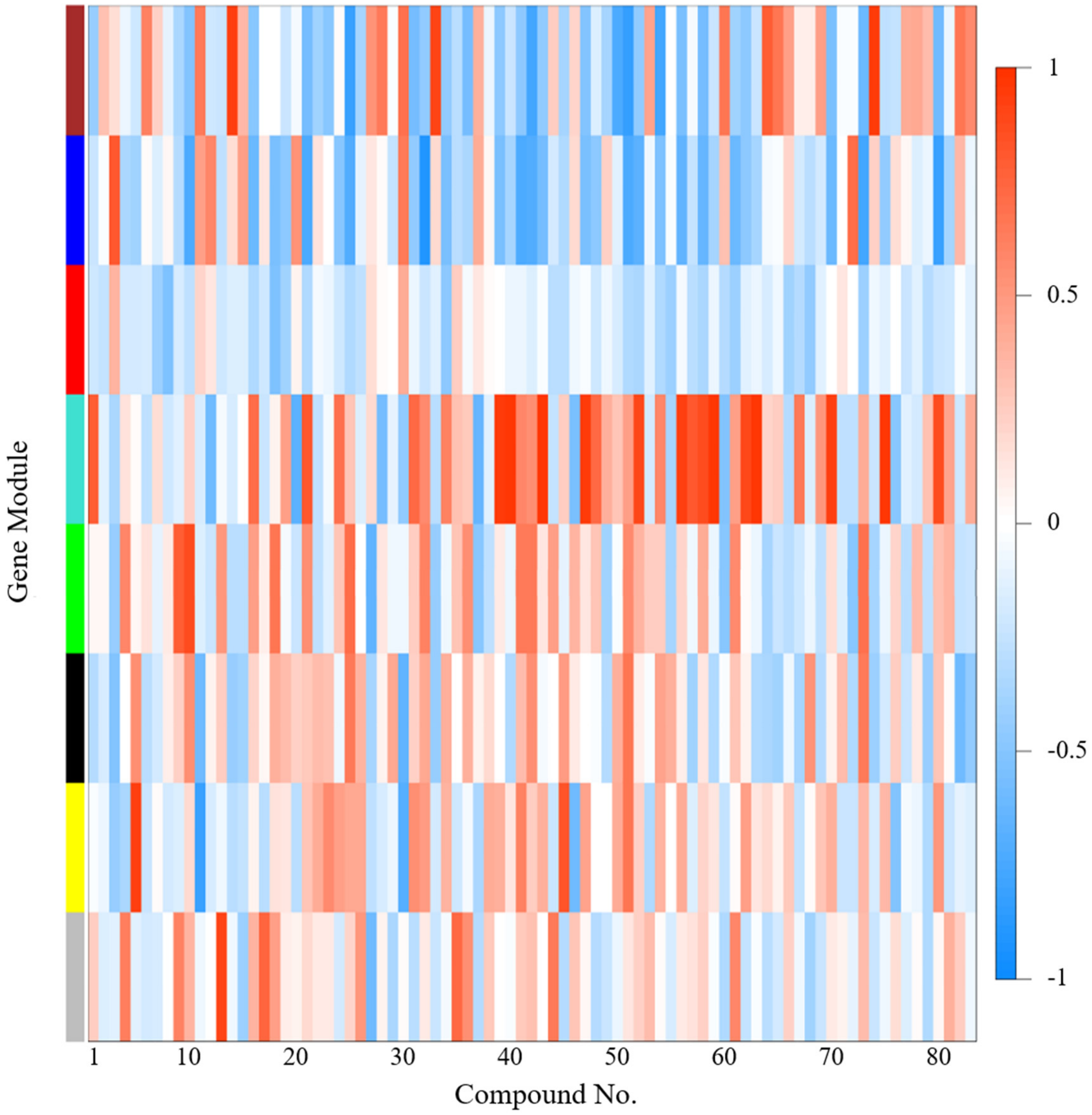

Figure S4. The correlation value among gene modules and volatiles. Volatiles were labeled by numbers consistent with Tables S2 and S3.

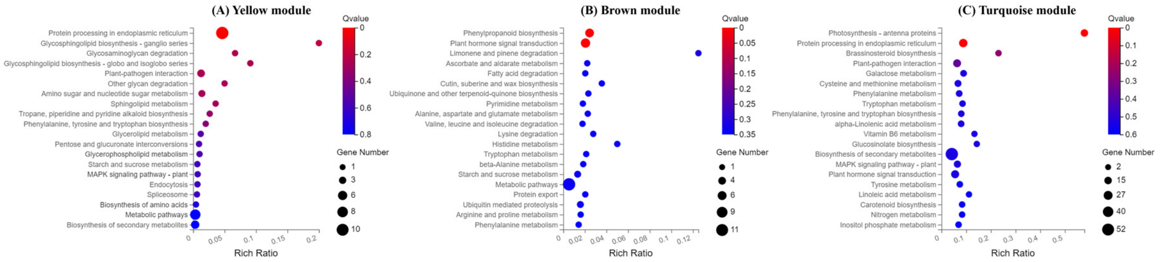

Figure S5. KEGG enrichment of three gene modules.
