# Supplementary material for: Effect of Climate on Volatile Metabolism in ‘Red Globe’ Grapes (Vitis vinifera L.) during Fruit Development
Source: Foods. 2022 May 16;11(10):1435. doi: 10.3390/foods11101435 (PMC9140514; doi:10.3390/foods11101435)
Supplement: Supplementary file 1 [file foods-11-01435-s001.zip › Table S1.pdf]

Table S1. Sequences and relative expression of validated genes (n = 3) *.

Gene ID	Gene Name	RNA seq Log_2FC (FC>2, p<0.01, US*vs*TS)					Primers	Sequence (5'-3')	Relative expression	S1	S2	S3	S4	S
		S1	S2	S3	S4	S5								
100253716	UBI						Forward	AATCCAGCGATACCATCGAC	-	-	-	-	-	-
							Reverse	CGAACCAGATGCAGAGTTGA	-	-	-	-	-	-
100264000	WRKY47	-2.32					Forward	GGAGCACCAAGGAGCAGTTA	TS	1.000±0.015	0.004±0.000	2.359±0.070	2.100±0.034	1.872±0.048
							Reverse	GGTGGTACAGGATTGAGGGA	US	0.308±0.017	0.838±0.049	1.799±0.025	1.749±0.087	2.078±0.067
100263252	REVEILLE 1	-2.65	-2.11		1.08		Forward	ACCAGTCCCCCACATCAGTA	TS	1.003±0.057	2.720±0.050	1.637±0.046	3.992±0.065	8.344±0.188
							Reverse	CGTTAAGACCGGGGATGGAG	US	0.104±0.013	0.965±0.122	1.036±0.028	5.089±0.365	4.705±0.113
100243201	LOB21		2.62	1.56			Forward	AGCTCCAACATGATCTCGCC	TS	1.001 ± 0.033	0.366 ± 0.007	0.218 ± 0.023	0.059 ± 0.008	0.043 ± 0.007
							Reverse	TTGATGGCCACAGGCTGATT	US	2.184±0.033	3.351±0.098	0.382±0.040	0.118±0.014	0.033±0.002
100255943	ERF003	-3.82	-2.34	-1.72			Forward	TTTCCTTTCGTCTTCTTCCA	TS	1.009±0.093	5.269±0.289	13.52±0.136	11.80±0.283	5.113±0.368
							Reverse	ATGTGCCGAGCCATATTCTA	US	0.027±0.003	0.181±0.018	5.813±0.314	7.179±0.808	9.672±0.493
100244353	ERF5		1.33	2.96			Forward	TTGTCGCAAACTTCAAGCCC	TS	1.001±0.027	0.550±0.018	0.259±0.018	0.467±0.021	0.360±0.002
							Reverse	AGATGCACAGTGAAGGTCGC	US	1.272±0.089	0.638±0.013	0.983±0.092	0.245±0.025	0.041±0.007
100255447	ERF62			2.24			Forward	CACAGCCCATGAAATACACG	TS	1.002±0.049	1.011±0.128	0.906±0.049	2.374±0.039	1.720±0.038
							Reverse	CCTGGTCCTGTTTCTCGGTA	US	0.643±0.054	2.204±0.248	4.429±0.057	1.190±0.201	2.108±0.197
100852860	bHLH36		-3.42	-6.02	-2.73	1.79	Forward	AAGGGTTCTGCTCTGGAGGT	TS	1.008±0.090	0.149±0.011	0.330±0.032	0.281±0.025	0.737±0.066
							Reverse	ATTTTAGCCCACCTGTGCAT	US	0.218±0.059	0.712±0.042	0.012±0.001	0.304±0.046	0.258±0.021
100266006	bHLH95		-3.31	-2.39	-1.50	-1.35	Forward	CGTTGTGGTGAGCATGTGTG	TS	1.010±0.100	0.109±0.009	0.559±0.041	1.766±0.036	5.633±0.496
							Reverse	TGGAGGAAATGTGGGCAGAC	US	0.023±0.004	0.101±0.007	0.152±0.015	0.559±0.048	1.516±0.019
100258060	HSFA-6b	3.02		1.01	-3.76		Forward	GTGGTCTCTTGGAGCAGAGC	TS	1.013±0.114	0.401±0.010	0.396±0.066	0.910±0.047	1.025±0.120
							Reverse	AGTGTTGAGCTGCCTGACAA	US	0.912±0.013	0.982±0.132	0.479±0.065	0.190±0.007	0.240±0.027
100245740	HSF30	2.88			-2.31		Forward	TCTTCACCAGGCTTTGCTCC	TS	1.001±0.034	1.121±0.165	0.964±0.050	1.459±0.114	0.794±0.125
							Reverse	AGTCGGTTGCTGGGTCTTCC	US	2.161±0.133	1.328±0.114	0.785±0.110	0.335±0.041	0.429±0.027
100258873	bHLH34	-1.14	1.19		-2.27		Forward	GCAGCCACCAAAGAATGAGC	TS	1.003±0.054	3.326±0.047	4.895±0.140	5.777±0.069	0.931±0.094
							Reverse	CACCCTTGACCTCTGAGCTG	US	0.435±0.114	6.314±0.961	5.605±0.090	0.932±0.053	1.446±0.188
100264647	ERF110				-4.86	-6.66	Forward	CGCCTGGCTTCTCCTTTACA	TS	1.003±0.056	2.497±0.210	0.323±0.040	0.707±0.060	0.231±0.028
							Reverse	TGCCGGAATATCTGCTCCAC	US	1.253±0.041	1.569±0.143	0.334±0.045	0.035±0.002	0.040±0.001
100256849	WRKY22	1.14	1.28		-1.83	-4.73	Forward	AGAGCACAACCACCCTATGC	TS	1.002±0.049	1.484±0.099	4.190±0.209	1.415±0.140	8.427±0.775
							Reverse	CACGCTTGTGCTTTGCTGTA	US	0.830±0.102	2.032±0.430	1.350±0.061	0.119±0.016	0.076±0.011
100246583	MYB-R	1.16			-1.32	-3.85	Forward	ACCTCGACACCTATCCCACA	TS	1.006±0.073	0.031±0.003	0.102±0.000	0.401±0.032	1.300±0.078

							Reverse	AGCCGCGCTCTATCAATGAA	US	0.043±0.001	ND	0.169±0.027	0.142±0.014	0.078±0.017
100261077	DnaJ	3.53	1.87	2.04	-1.46	1.85	Forward	ACGAACTCAATGGTGGACAG	TS	1.006±0.079	1.239±0.046	2.833±0.270	7.490±0.257	1.785±0.145
							Reverse	ACTGGAAACAGCCACAAATC	US	7.572±0.397	5.891±0.207	9.484±0.359	2.223±0.098	10.353±0.627
100252547	PCC13	3.95	2.62	1.74	2.26	2.17	Forward	CACGAACCCACTTCTCATAG	TS	1.003±0.052	0.163±0.016	0.092±0.010	0.079±0.003	0.061±0.001
							Reverse	AGCTCGCTCATACAAATACG	US	1.558±0.011	1.112±0.142	0.326±0.018	0.277±0.027	0.432±0.052

[*] US: Urumqi sample; TS: Turpan sample; FC: fold change; S: stage.
