# Supplementary material for: Effect of Climate on Volatile Metabolism in ‘Red Globe’ Grapes (Vitis vinifera L.) during Fruit Development
Source: Foods. 2022 May 16;11(10):1435. doi: 10.3390/foods11101435 (PMC9140514; doi:10.3390/foods11101435)
Supplement: Supplementary file 1 [file foods-11-01435-s001.zip › Table S2.pdf]

Table S2. Detected volatiles and their structures.

No.	Retention Time	Compound	Structure
1	3.66	n-Hexane	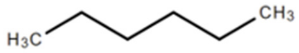
2	5.50	3-Methylpentane	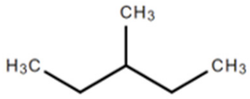
3	7.50	Arginine	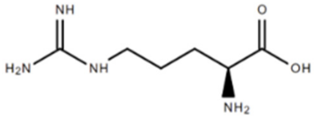
4	7.61	Hexanal	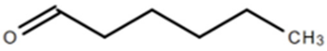
5	7.84	Undecane	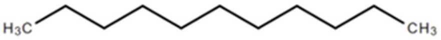
6	10.20	β-Myrcene	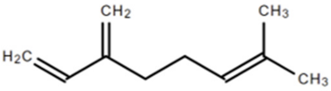
7	11.20	D-Limonene	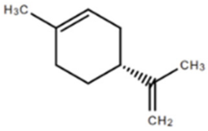
8	11.59	2-Heptynol	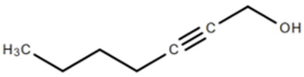
9	11.68	Dihydrocarveol	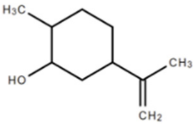
10	12.07	(E)-2-Hexenal	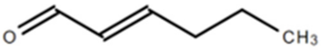

11	12.50	(E,E)-2,4-Nonadienal	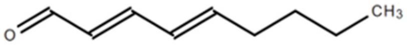
12	12.80	4,6-Dimethyldodecane	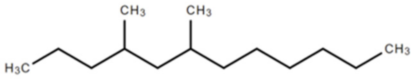
13	13.30	Styrene	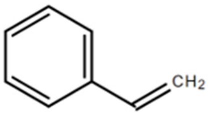
14	14.12	2-Octanone	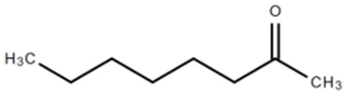
15	14.40	Tridecane	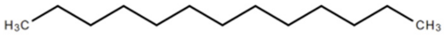
16	15.27	(E)-Hept-2-enal	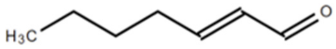
17	16.21	1-Hexanol	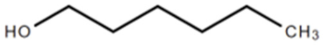
18	17.25	Nonanal	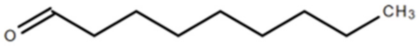
19	17.58	(E,E)-2,4-Hexadienal	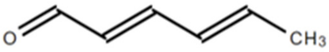
20	17.71	2-Hexen-1-ol	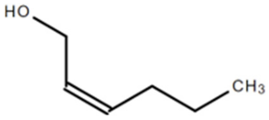
21	18.22	(E)-2-Octenal	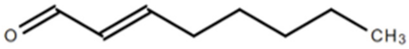
22	18.37	2,6,10-Trimethyldodecane	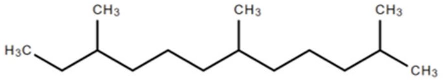

23	18.52	2,6,11-Trimethyldodecane	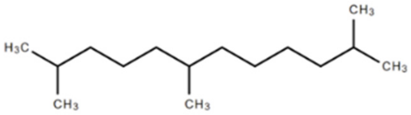
24	18.88	3-Octenol	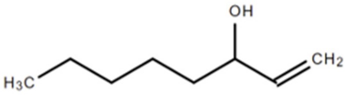
25	19.28	2,4-Heptadienal	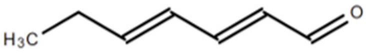
26	19.84	2,7,10-Trimethyldodecane	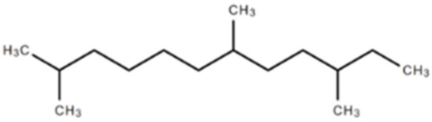
27	19.98	4-Ethylcyclohexanol	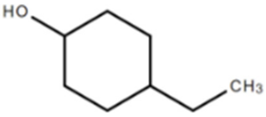
28	20.05	Decanal	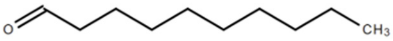
29	20.78	Benzaldehyde	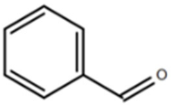
30	20.98	2-Nonenal	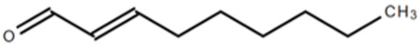
31	21.36	Linalool	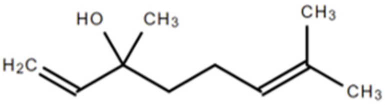
32	22.22	Hexadecyl chloride	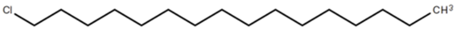
33	22.27	(E,Z)-2,6-Nonadienal	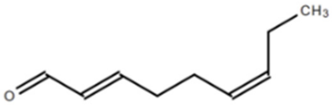
34	22.41	6-Methyltridecane	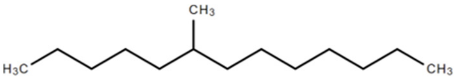

35	23.33	2,6,10,15-Tetramethylheptadecane	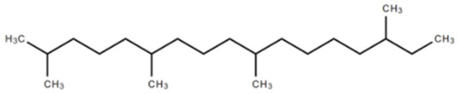
36	23.60	3-Heptylacrolein	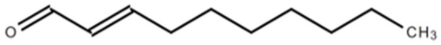
37	23.74	Benzeneacetaldehyde	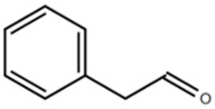
38	24.45	1-Iodoeicosane	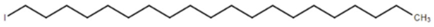
39	24.53	α-Cadinene	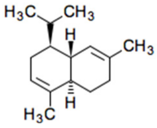
40	24.88	α-Terpineol	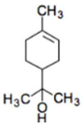
41	24.98	Germacrene D	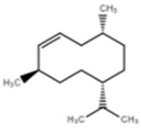
42	25.13	(6E)-8-Hydroxylinalool	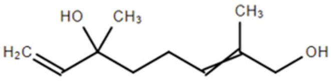
43	25.36	α-Muurolene	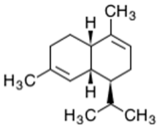
44	25.86	Azulene	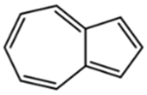
45	25.86	Naphthalene	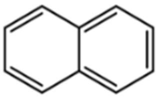
46	26.06	2-Undecenal	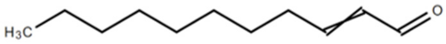

47	26.11	δ-Amorphene	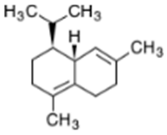
48	26.43	(E,E)-2,4-Decadienal	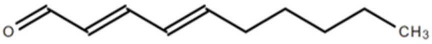
49	26.50	Methyl N-hydroxybenzenecarboximidoate	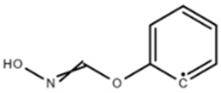
50	27.45	2,5-Dimethylbenzaldehyde	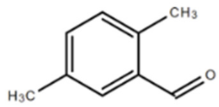
51	27.58	β-Damascenone	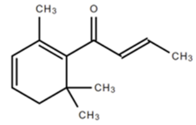
52	28.31	(Z)-Geranylacetone	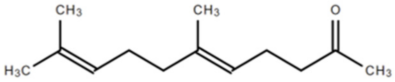
53	28.40	(E)-2-Dodecenal	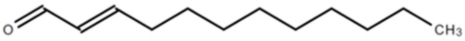
54	28.62	Texanol	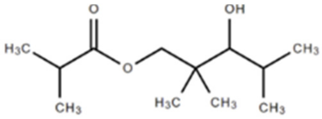
55	28.70	Kodaflex txib	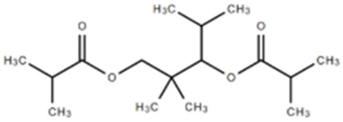
56	29.55	α-Calacorene	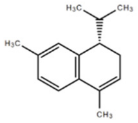
57	29.66	1-Octadecyne	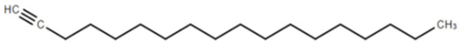
58	30.02	β-Ionone	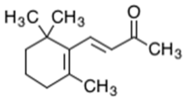

59	30.80	Phytol	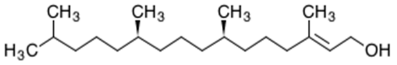
60	30.86	(E)-2-Hexenoic acid	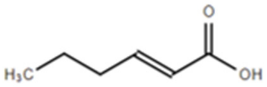
61	31.10	1,6,6-Trimethyl-7-(3-oxobut-1-enyl)-3,8-dioxatricyclo [5.1.0.0(2,4)]octan-5-one	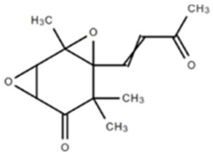
62	31.40	Glutaconic anhydride	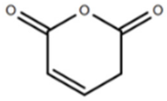
63	32.35	Junenol	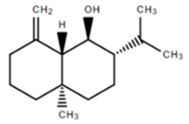
64	32.59	3-Hydroxy-4-methoxybenzonitrile	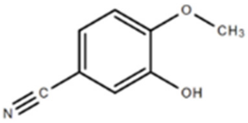
65	33.01	Nonadecane	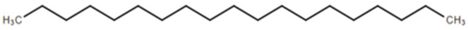
66	33.85	Ethyl cinnamate	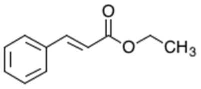
67	34.22	Olivetol	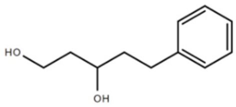
68	34.60	Nonanoic acid	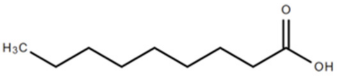
69	35.27	Methyl palmitate	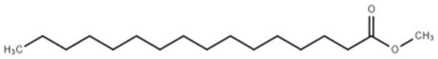
70	35.48	Cadalin	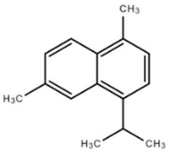

71	36.57	N,N-Diethyl-P-Toluamide	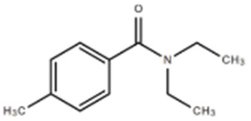
72	37.19	2,4-Di-tert-butylphenol	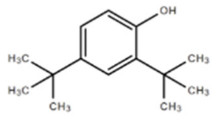
73	37.64	Dihydroactinidiolide	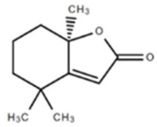
74	37.72	(-)-Copalol	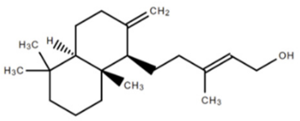
75	38.70	Coumaran	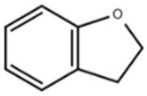
76	39.40	β-1,5-Dibenzoyl-2-deoxy-ribofuranose	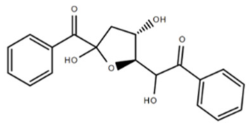
77	39.53	Indole	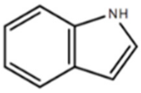
78	40.05	Dodecanoic acid	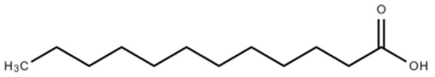
79	40.91	Diisobutyl phthalate	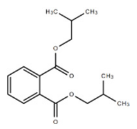
80	41.44	Vanillin lactoside	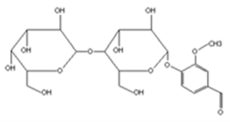
81	43.32	Butyl octyl phthalate	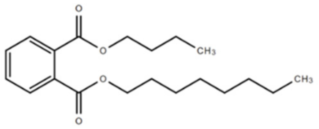
82	44.08	Imidazole, 2-amino-5-[(2-carboxy)vinyl]-	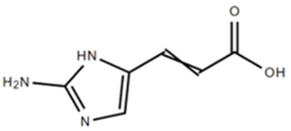

83	46.40	n-Hexadecanoic acid	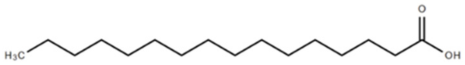

[*] Structures could be referred to websites: www.ichemistry.cn and www.kegg.jp.
