# Supplementary material for: Effect of Climate on Volatile Metabolism in ‘Red Globe’ Grapes (Vitis vinifera L.) during Fruit Development
Source: Foods. 2022 May 16;11(10):1435. doi: 10.3390/foods11101435 (PMC9140514; doi:10.3390/foods11101435)
Supplement: Supplementary file 1 [file foods-11-01435-s001.zip › Table S4.pdf]

Table S4. Alignments of RNA-sequencing reads*.

Sample	Total Raw Reads (M)	Total Clean Reads (M)	Total Clean Bases (Gb)	Total Mapping (%)	Uniquely Mapping (%)
TS-S1-1	45.57	44.39	6.66	89.86	87.74
TS-S1-2	45.57	44.45	6.67	89.06	86.53
TS-S2-1	45.57	44.37	6.66	88.67	86.11
TS-S2-2	45.57	44.57	6.69	88.64	86.13
TS-S2-3	47.33	44.91	6.74	87.35	84.75
TS-S3-1	45.57	44.28	6.64	87.63	84.82
TS-S3-2	45.57	44.37	6.66	88.78	86.17
TS-S3-3	45.57	44.45	6.67	88.65	85.89
TS-S4-1	45.57	44.38	6.66	89.11	86.53
TS-S4-2	45.57	44.49	6.67	89.19	86.69
TS-S4-3	45.57	44.58	6.69	88.94	86.35
TS-S5-1	45.57	44.38	6.66	90.1	87.74
TS-S5-2	44.14	42.98	6.45	89.47	87.03
TS-S5-3	45.57	44.24	6.64	87.11	82.92
US-S1-1	45.57	44.58	6.69	91.23	88.79
US-S1-3	45.57	44.6	6.69	89.97	87.74
US-S2-1	47.33	44.99	6.75	85.91	82.99
US-S2-2	41.12	40.25	6.04	77.33	73.58
US-S2-3	45.57	44.25	6.64	85.06	82.49
US-S3-1	45.57	44.36	6.65	87.62	84.96
US-S3-2	45.57	44.24	6.64	88.24	85.69
US-S3-3	45.57	44.3	6.64	86.74	83.59
US-S4-1	45.57	44.36	6.65	85.74	83.26
US-S4-2	45.57	44.38	6.66	85.28	82.67
US-S4-3	45.57	44.25	6.64	87.09	84.38
US-S5-1	45.57	44.18	6.63	86.12	83.67
US-S5-2	45.57	44.24	6.64	85.45	82.83
US-S5-3	45.57	44.08	6.61	85.95	83.33

* TS: Turpan sample; US: Urumqi sample; S: stage
